# Translational assessment instruments for preclinical to first-in-human decision-making: a scoping review

**DOI:** 10.1186/s12967-026-08604-1

**Published:** 2026-07-13

**Authors:** Marco Straccia, David Mawufemor Azilagbetor, Celean Camp, Guilherme S. Ferreira, Maria Laura Garcia-Bermejo, Annalisa Gastaldello, Kurinchi Gurusamy, Francesco Pappalardo, Merel J. Ritskes-Hoitinga, Fabrizio Rossi, Danilo Tagle, Martin Wehling, Francesca Pistollato

**Affiliations:** 1 FRESCI by Science&Strategy SL, Barcelona, Spain; 2 Institute for Biomedical Ethics, University of Basel, Basel, Switzerland; 3 Replacing Animal Research, Nottingham, UK; 4 Utrecht University, Utrecht, The Netherlands; 5 European Infrastructure for Translational Medicine (EATRIS), Amsterdam, The Netherlands; 6 Ramón y Cajal Health Research Institute (IRYCIS), Madrid, Spain; 7 European Commission, Joint Research Centre (JRC), Ispra, Italy; 8 Division of Surgery and Interventional Science, University College London (UCL), London, UK; 9 Department of Drug and Health Sciences, University of Catania, Catania, Italy; 10 Aarhus University, Aarhus, Denmark; 11 Office of Special Initiatives, National Center for Advancing Translational Sciences (NCATS), National Institutes of Health, Bethesda, MD USA; 12 Clinical Pharmacology, Heidelberg University, Mannheim, Germany; 13 Humane World for Animals International, Brussels, Belgium

**Keywords:** Translational medicine, First-in-human, Preclinical research, Translatability, Evidence-to-decision, Uncertainty, Interoperability, Risk of bias, Decision support, Scoping review

## Abstract

**Background:**

Preclinical evidence used to support first-in-human (FIH) decisions is often assembled through isolated assessment tools that improve individual parts of translational practice but do not preserve assumptions, uncertainty, and decision intent across discovery-to-clinic handoffs. We therefore reviewed structured translational assessment instruments designed to inform preclinical-to-human decision-making and examined whether the published landscape already contains the components needed for decision-grade evidence integration.

**Main body:**

We conducted a scoping review informed by PRISMA-ScR principles to identify peer-reviewed publications describing structured assessment instruments intended to support preclinical-to-clinical translation decisions. Searches were performed in PubMed and complemented by citation snowballing. Nineteen distinct instruments were identified and mapped across the preclinical-to-FIH pathway. The landscape showed clear strengths in model-selection support, reporting quality, risk-of-bias control, and mechanistic structuring of translational claims. However, recurrent limitations were also evident. Few instruments explicitly benchmark performance against downstream human outcomes. Uncertainty associated with evidence generated at one stage is often not explicitly propagated to later stages of the research and decision-making process. Outputs are rarely designed as reusable governance artefacts with explicit context of use, decision owner, breach conditions, and update triggers. The ecosystem is therefore stronger in decision-grade evidence production than in decision-grade evidence integration. Based on this synthesis, we derive a conceptual interoperability framework in which evidence-facing outputs remain source-traceable and uncertainty-aware, while decision-facing outputs are released only within defined governance gates. Within such a framework, AI is most defensible as a constrained support layer for retrieval-grounded extraction, normalization, and interoperability, rather than as an autonomous decision-maker.

**Short conclusion:**

Current translational practice is limited less by the absence of assessment instruments than by weak interoperability between them. A staged, uncertainty-aware workflow that preserves assumptions and decision logic across handoffs could improve the auditability and reuse of preclinical evidence for FIH governance. Whether such workflows improve predictive performance or downstream clinical decision quality remains to be tested prospectively.

**Supplementary Information:**

The online version contains supplementary material available at https://doi.org/10.1186/s12967-026-08604-1.

## Background

Drug development remains slow, expensive and characterised by high attrition across clinical phases [1, 2]. Despite sustained investment in translational science, preclinical findings frequently fail to predict clinical efficacy or safety in humans, contributing to prolonged timelines and late-stage programme failure [2–6]. Many instruments now exist to improve model choice, study rigour and programme-level translational assessment [7,8], but they are often implemented as isolated instruments rather than as an integrated decision workflow that produces standardised governance outputs. Governance of preclinical to first-in-human (FIH) translation is distributed across multiple institutions and decision levels rather than assigned to a single body. Relevant decision-makers include scientific and translational teams, sponsor or portfolio-governance bodies, funders, ethics and institutional quality bodies, and regulatory authorities; their principal roles and decision contexts are summarised in Table 1. As a result, key assumptions and uncertainty are rarely carried forward across discovery to FIH handoffs, and forces teams to repeatedly rebuild rationales rather than accumulating a continuous, auditable evidence thread.

**Table 1:** Governance stakeholders and decision contexts in preclinical-to-FIH translation. The table identifies representative governing bodies and potential decision-makers, together with the principal decisions they may inform. The examples are illustrative rather than exhaustive; formal responsibilities, institutional terminology, and final decision authority vary by jurisdiction, organisation, development programme, and context of use

Stakeholder Group	Decision Makers	Decision Context
Research & Discovery	Academic & Industry Investigators	Study Design & Model Selection: Choosing the right experimental system to test a hypothesis while ensuring rigorous design.
	Translational Pharmacologists & Biologists	Biomarker & Assay Validation: Ensuring read-outs are clinically relevant.
Strategic & Financial	Pharma/Biotech Portfolio Managers	Portfolio Management (Go/No-Go): Deciding which assets to advance to the next phase to minimise attrition.
	Venture Capitalists & Funders	Due Diligence & De-risking: Evaluating the robustness of external assets or grant proposals.
Governance & Oversight	Ethics Committees	Harm-Benefit Analysis: Determining if the scientific value justifies animal use.
	Institutional Quality Managers / Process Owners	Quality Assurance: Implementing culture change and maintaining data integrity standards.
Regulatory & Policy	Regulators (FDA, EMA, etc.)	Evidence Review & Method Qualification: Assessing safety/efficacy packages for preclinical-to-FIH development pathway (IND/CTA enabling package).
	Policy Makers & Consortia	Standard Setting: Defining acceptable evidence thresholds.

Despite their availability, uptake appears heterogeneous and implementation is often resource- and expertise-limited, which pushes many instruments toward retrospective assurance rather than prospective decision support [7]. This fragmentation increases curation burden and can harden epistemic silos, while providing limited assurance that evidence packages will be decision-grade for the specific regulatory, ethical, or portfolio context in which they are used. Given this fragmented landscape, there is a pressing need for a system-level approach based on an integrated chain of decision-making supporting tools [8–10] that can be easily adopted by end-users in the drug discovery pipeline, helping to bridge the gap from early discovery through FIH use. An AI-assisted workflow approach could reduce duplicated curation, improve auditability, and standardise how uncertainty is carried forward. Whether such integration improves clinical attrition is an empirical question that should be tested prospectively rather than assumed.

Beyond efficiency, the central limitation is decision credibility. Many frameworks improve documentation, rigour or relevance, yet do not fully specify the context of use (CoU) in which outputs are intended to support governance decisions. And “decision-grade” should mean an evidence package with an explicit CoU, uncertainty profile, and pre-specified progression logic that a governance body can deliberate without reconstructing assumptions. Without explicit decision thresholds, uncertainty representation and reusable decision artefacts, preclinical evidence can be scientifically defensible but still poorly calibrated for irreversible calls such as FIH exposure, portfolio prioritisation or programme termination [11–13].

Many published instruments already address important parts of translational practice, including model selection, study rigor, reporting quality, mechanistic plausibility, and progression logic. The unresolved problem is not simply the absence of tools, but the absence of an interoperable workflow that preserves assumptions, uncertainty, and decision intent across the preclinical-to-FIH pathway. The objective of this scoping review was therefore to identify and classify structured translational assessment instruments intended to inform preclinical to human decision-making, compare their dominant functions and evidence outputs, and derive a conceptual interoperability framework for reusable, uncertainty-aware evidence-to-decision (E2D) workflows.

## Review methods and evidence base

This work was conducted as a scoping review informed by PRISMA-ScR principles to map, classify, and compare structured assessment instruments intended to inform preclinical-to-clinical translation decisions. Because the objective was to characterize the published ecosystem of tools and frameworks rather than to estimate pooled effect sizes, we used a scoping rather than a systematic review approach.

Because terminology was not used consistently across the included publications, we developed operational definitions for the purposes of data extraction and comparative synthesis. These definitions specify how each term was interpreted in the present review and should not be understood as claims that the authors of the original publications used identical terms to denote identical constructs. Supplementary Information file (Suppl. Table 1) reports each term, its operational meaning in this review, its principal decision level and timing, and its relationship to adjacent concepts.

We searched PubMed for English-language peer-reviewed journal articles published between 1 January 2006 and 1 December 2025 and complemented database retrieval by citation snowballing. The search strategy combined three concept blocks: preclinical models, translational value or validity, and structured assessments or decision-support instruments. We included publications proposing, validating, or applying a structured assessment instrument, framework, checklist, scoring system, or algorithm intended to inform preclinical-to-human translation decisions in therapeutic development. Instruments could address in vivo, in vitro, in silico, or new approach methodology evidence, provided that their stated purpose related to translation to humans. We excluded instruments intended exclusively for human clinical trials without explicit adaptation for preclinical evidence, narrowly disease-specific tools without an explicit cross-programme claim, and regulatory chemical hazard frameworks whose decision context differs materially from therapeutic development. The full search strategy is provided in the Supplementary Information file.

Records retrieved from PubMed and citation snowballing were deduplicated and screened by two reviewers (M.S. and F.R.) using a web-based screening workflow. Disagreements at title/abstract or full-text stage were resolved by discussion. In the current review dataset, 1,877 records were identified, 1,846 remained after deduplication, 174 full-text articles were assessed for eligibility, and 19 distinct instruments were included in the qualitative synthesis. For each included instrument, we extracted the tool identity, intended use case, assessment domains covered, implementation requirements, and validation status. We then conducted a narrative synthesis supported by conceptual mapping across workflow stage, epistemic focus, and decision utility.

AI-assisted tools were used only to support literature retrieval refinement and language editing. Any such output was treated as non-authoritative, verified against the source literature by the authors, and manually revised before inclusion. No AI system was used to generate study data, perform unsupervised final classification of instruments without author verification, or make editorial decisions about manuscript content. The full search strategy, PRISMA flow diagram, and supplementary extraction details are provided in the Supplementary Information file.

## Landscape of translational assessment instruments

We identified 19 instruments differing in intended decision use, evidence inputs, assessment methodology and degree of uncertainty representation. To transparently report this heterogeneity, we mapped each instrument in a descriptive manner in Table 2 (also in Suppl. Table 3). The instruments generate different kinds of decision objects. Some tools primarily produce qualitative outputs, including checklists, reporting standards, quality-system declarations, planning records, or governance documentation. PREPARE [13], DEPART [14], ARRIVE 2.0 [15, 16], GOT-IT [17], EQIPD [18], 3Vs [19], SYRCLE RoB [20], the Replacement Checklist [21], and NAMs-SCF [8] fall within this group. Other instruments generate semi-quantitative structured judgements, such as colour-coded matrices, certainty ratings, domain concern ratings, or annotated mechanistic pathways; examples include AMQA [22], RAA [23], GRADE4Animals [24], and PATH [25]. A smaller subset produces numerical, performance-based, probability-like, or economic outputs, including AMF [26], FIMD [12, 27], the Translatability Score [28, 29], OTP [30], PoPS [10], and D-TF [31]. This distinction matters because the risk of false precision varies across the landscape. It becomes greatest when weighted domain ratings, association scores, probabilities, or economic estimates are interpreted in isolation from their assumptions, missing evidence, indirectness, and validation status.

**Table 2:** Instrument-level map of translational tools. Instruments are presented in chronological order. A detailed version is in Supplementary Table 3

Instrument	Context of Use	Evidence structured	Output type / decision output	Main uncertainty to surface
2009 - Animal Model Framework (AMF)	Assess animal-to-human safety translation	Retrospective non-clinical and Phase I adverse-event data	**Quantitative**: sensitivity, specificity, predictive capacity	Endpoint comparability, historical-data bias, animal–human concordance
2009 (2017) - Translatability Score	Portfolio risk assessment and go/no-go support	Project data from in vitro, in vivo, genetics, biomarkers, clinical need, IP and trials	**Quantitative**: weighted score plus stopper logic	Weighting assumptions, disease-area calibration, false precision
2014 - SYRCLE’s Risk of Bias (RoB) Tool	Assess internal validity of animal studies	Randomisation, allocation, blinding, attrition, reporting and other bias domains	**Qualitative/categorical**: low, high or unclear risk of bias	Incomplete reporting, reviewer judgement, no direct predictive validity
2017 - DEPART (Design and Execution of Protocols)	Improve design, execution and analysis quality	Study objectives, model rationale, reagents, bias controls, statistical plans	**Qualitative**: checklist compliance across design, conduct and analysis	Implementation fidelity, local adherence, no predictive output
2018 - GRADE4Animals	Grade certainty of preclinical animal evidence	Systematic-review evidence, effect estimates, risk of bias, indirectness	**Semi-quantitative**: high, moderate, low or very low certainty	Downgrading judgement, publication bias, indirectness to humans
2018 - PREPARE (Planning Research and Experimental Procedures)	Improve pre-experimental planning	Hypothesis, literature search, ethics/legal constraints, facility coordination	**Qualitative**: planning checklist/action plan	Compliance burden, variable uptake, no translatability estimate
2019 - Framework to Identify Models of Disease (FIMD)	Select fit-for-purpose disease models	Human indication and model evidence across disease-relevance domains	**Quantitative**: 0–100 score, radar plot, validation level, missing-data uncertainty	Domain weighting, missing evidence, publication bias, biological indirectness
2020 - Animal Research: Reporting of In Vivo Experiments (ARRIVE) 2.0	Improve reporting transparency and reproducibility	Essential reporting items: study design, sample size, randomisation, blinding, outcomes	**Qualitative**: completed reporting checklist	Reporting completeness does not guarantee study quality or predictivity
2021 - Animal Model Quality Assessment (AMQA)	Support animal model selection and harm–benefit analysis	Internal/external team evidence on target biology, model physiology, disease and pharmacology	**Semi-quantitative**: red/amber/green matrix plus narrative rationale	Boundary subjectivity, mitigation quality, limited predictive benchmarking
2021 - Clinical Relevance Assessment of Animal Preclinical Research (RAA) Tool	Assess clinical relevance of animal preclinical research	Study design, bias controls, reproducibility, external validity and transparency	**Semi-quantitative**: domain concern ratings and overall relevance judgement	Context dependency, expert judgement, limited outcome calibration
2021 - Guidelines On Target Assessment (GOT-IT)	Identify target-assessment red flags and critical-path actions	Project data on linkage, safety, microbial targets, strategic and technical feasibility	**Qualitative**: project-specific critical path, milestones, go/no-go points	Data quality, feasibility assumptions, stakeholder-dependent prioritisation
2021 - Open Targets Platform (OTP)	Prioritise target–disease associations	Genetics, somatic mutations, pathways, drugs, expression, animal models, text mining	**Quantitative**: association score, tractability and safety assessments	Source heterogeneity, evidence weighting, association not equal to clinical success
2021 - EQIPD Quality System	Ensure preclinical data are fit for intended use	Research-unit processes, study plans, data integrity, governance structures	**Qualitative**: quality-system declaration/certification	Implementation burden, audit variability, no direct predictive validity
2021–3Vs Framework (3Vs)	Assess scientific suitability before harm–benefit review	Protocol, model choice, intended inference space and experimental design	**Qualitative**: construct → internal → external validity judgement	Sequencing judgement, qualitative gate, no quantified predictive performance
2022 - Decision-Theoretic Framework (D-TF)	Estimate value of predictive validity for R&D productivity	Predictive validity, throughput, selection thresholds, base rates, costs and profits	**Quantitative**: PPV and NPV consequences	Base-rate assumptions, economic parameters, model sensitivity
2022 - NAMs Scientific Confidence Framework (NAMs-SCF)	Establish confidence in NAMs for regulatory use	Protocol, human biological relevance, technical reliability, data integrity	**Qualitative**: fitness-for-purpose confidence determination	CoU specificity, regulatory acceptance, limited cross-context generalisability
2023 - Probability of Pharmacological Success (PoPS)	Support commit-to-clinical decisions	Exposure–response models, translational pharmacology, safety data and success criteria	**Quantitative**: probability of pharmacological success	Model assumptions, virtual-population uncertainty, success-criteria selection
2024 - Preclinical Assessment for Translation to Humans (PATH)	Justify early-phase trial rationale	Mechanistic chain evidence across animal, in vitro and human data	**Semi-quantitative**: annotated pathway, confidence ratings, critical breaches	Confidence judgement, source bias, causal-link incompleteness
2025 - Replacement Checklist	Document replacement due diligence and 3Rs compliance	Research question, alternative-method searches, databases, experts and AI-supported sources	**Qualitative**: auditable checklist for replacement justification	Search completeness, local interpretation, no predictive translatability output

This mapping highlights that translational assessment is strongest when framed as a set of complementary decision functions, rather than as a search for a universal “translatability score”. Reporting and quality-system tools provide procedural assurance; model-selection tools provide model-credibility rationales; mechanistic and target-assessment tools provide causal or prioritisation logic; and translation-facing tools provide readiness, probability, or progression signals. These outputs can support FIH governance when their underlying assumptions and uncertainties remain visible. Quantification can aid prioritisation, scenario testing, and portfolio comparison. Where predictive validity has been explicitly benchmarked against downstream human outcomes, quantified outputs may support stronger claims about clinical translatability; in other settings, they are best treated as structured decision support rather than direct measurements of translational likelihood.

### Positioning tools across preclinical-to-FIH development pathway

**Fig. 1:**
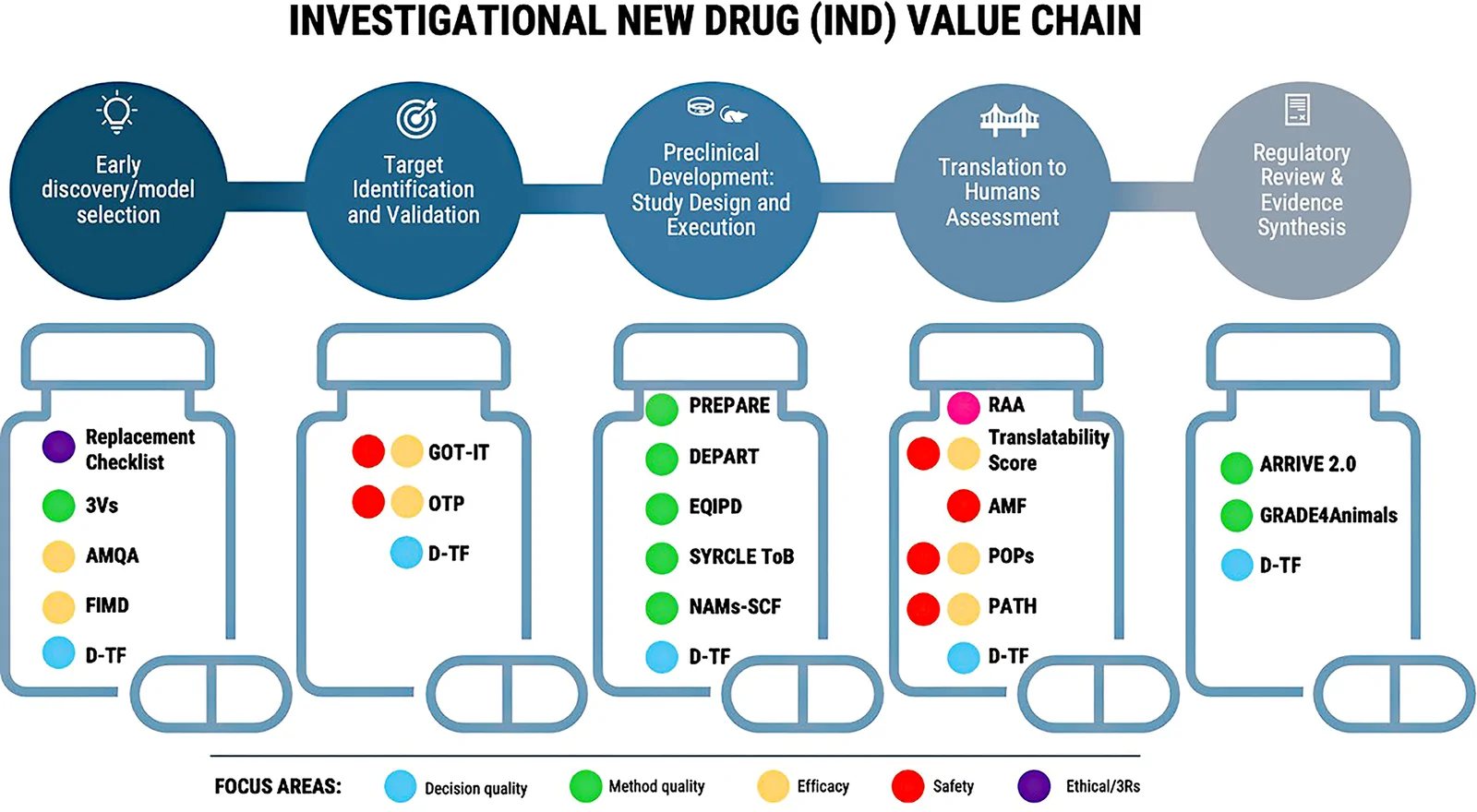
Where translational instruments sit in the discovery-to-FIH workflow. Instruments are positioned by their most common point of activation; many span multiple stages. Colour labels indicate the dominant focus of the instrument’s output (decision quality, method quality, efficacy, safety, ethical/3Rs). (1) Early Discovery / Model Selection: The foundational stage where researchers identify, evaluate, and select the most appropriate experimental systems to serve as surrogates for human biology and disease. The goal is to establish Construct Validity and Face Validity by ensuring the chosen model accurately reflects the human condition’s aetiology, pathophysiology, and symptomatology. Selecting a poorly predictive model at this stage is a primary driver of downstream translational failure. (2) Target Identification and Validation: The process of discovering a novel molecular entity (such as an enzyme or receptor) linked to a disease process, and generating rigorous evidence that pharmacological modulation of this target will yield a therapeutic benefit in a defined patient population. Before designing molecules, researchers must confirm that the target is causally linked to the disease, “druggable,” and lacks severe safety liabilities (on-target toxicity). It also involves evaluating the competitive landscape and intellectual property viability. (3) Preclinical Development: Study Design and Execution: The operational phase where experiments on the selected models are planned, conducted, and documented to generate robust data on a therapeutic candidate’s efficacy and safety prior to human testing. The primary focus here is maximizing Internal Validity by minimizing systematic errors and biases. This requires meticulous attention to experimental hygiene, including proper randomization, blinding, sample size calculation, and transparent reporting. (4) Translation to Humans Assessment: The synthesis and evaluation of accumulated preclinical pharmacological, toxicological, and mechanistic data to predict clinical efficacy and safety, directly informing the critical “Go/No-Go” decision for first-in-human (Phase I) trials. This stage translates dynamic responses from the model to the patient. It requires estimating the probability of success by integrating uncertainties around drug potency, exposure, safety margins, and the relevance of biomarkers. (5) Regulatory Review & Evidence Synthesis: The formal, systemic aggregation and critical appraisal of a body of preclinical evidence to support high-stakes decisions, such as Investigational New Drug (IND) approvals by regulatory agencies (FDA/EMA), clinical trial authorizations, or ethical committee harm-benefit analyses. This phase relies on systematic reviews and meta-analyses to determine the aggregate certainty, robustness, and clinical relevance of the data. It acts as the final gatekeeper before exposing human subjects to novel interventions

We then positioned the 19 assessment instruments along the preclinical-to-FIH pathway (Fig. 1), and we observed a clear pattern where early discovery phase concentrates those tools that are focused on the reduction of the biological and target risk (D-TF [31], FIMD [12, 27], AMQA [22], OTP [30], GOT-IT [17]) and those formalising scientific suitability and ethical legitimacy (3Vs [19], Replacement Checklist [21]). Following, preclinical development stage concentrates instruments (PREPARE [13], DEPART [14], EQIPD [18], SYRCLE RoB [20], NAMs-SCF [8]) that reduce method and execution risk, reflecting the operational need to generate evidence that is both reproducible and ready to be synthesised. Translation-to-humans is populated by tools that explicitly integrate evidence into a programme-level judgement (AMF [26], PATH [25], Translatability Score [28, 29], PoPS [10], RAA [23]), while regulatory review and evidence synthesis rely on reporting completeness and structured grading of certainty (ARRIVE 2.0 [15, 16] and GRADE4Animals [24]).

When positioned along the preclinical-to-FIH development pathway (Fig. 1), the instruments landscape implies a staged operating model: (a) upstream instruments constrain model/target choice; (b) execution-stage instruments stabilise evidence integrity; and (c) translation-facing instruments convert evidence into committee-deliverable decision outputs. Operationally, this analysis clarifies assessment instruments and decision ownership: early discovery tools primarily support target/model selection decisions by scientific teams; execution-stage instruments support quality owners and reviewers by stabilising evidence integrity; and translation-to-humans tools support governance bodies by converting evidence into progression-relevant confidence statements and explicit breach conditions.

While there is some overlap between tools (Table 2; Fig. 1), the translation assessment instruments operate at different granularities (study, model, programme and portfolio) and produce outputs with varying decision-making potential. This creates a need for an interoperable workflow that preserves the full decision-relevant evidence thread across stages, including the translational claim, context of use, evidence sources, methodological quality, assumptions, uncertainty, decision criteria, decision ownership, breach conditions, and update history. We emphasise claims and uncertainty because these interpretive elements are particularly vulnerable to loss when a detailed assessment is reduced to a final score, category, or narrative conclusion. The more quantitative tools (e.g., FIMD, Translatability Score, AMF, OTP, PoPS, D-TF) can support prioritisation and scenario testing, but they typically depend on assumptions (weights, model structure, data completeness) that must be transparent to avoid false precision. Qualitative and semi-quantitative tools (e.g., SYRCLE RoB, PREPARE/DEPART, PATH, RAA) are often more robust under sparse data conditions and better aligned with committee-based governance, but they can be harder to aggregate across portfolios of evidence.

This initial mapping suggests a practical operating model where rather than searching for a universal “translatability score”, organisations may benefit from a staged toolkit that (i) improves model and target choices early, (ii) enforces rigorous design and documentation during preclinical execution, and (iii) integrates evidence into explicit translational claims before FIH decisions.

### Why and When to use the existing translational assessment instruments

We propose a higher-level taxonomy that complements the process mapping (Table 3) by classifying each instrument by (a) its dominant conceptual focus, and (b) the primary validity gap it is designed to mitigate. This taxonomy helps to navigate the end user of these instruments towards a specific decision context.

**Table 3:** Construct/external validity is the most broadly shared decision target across tool families, whereas internal validity/RoB control and replacement governance are handled by specialised instruments that typically must be chained into integrated translational workflows rather than used standalone. Predictive performance is explicitly foregrounded in only a subset, underscoring a gap between relevance assessment and quantified predictivity.

Dominant conceptual class	Instruments	Epistemic focus			
		**Construct and External Validity**	**Internal Validity & Risk of Bias Reduction**	**Predictive Validity and Performance**	**Go/No-Go Decision Making & Portfolio Progression**
		Is the disease phenotype/symptomatology consistent with the human condition? Are the findings consistent across different conditions, laboratories, or model variations?	Was the study conducted to minimise bias? Are the results reproducible?	Are the molecular/mechanistic targets conserved? Do reference drugs (positive controls) behave as expected? What is the CoU?	Can we commit resources and ethically proceed? How do we allocate capital across options over time?
Model-Centric	Framework to Identify Models of Disease (FIMD)[12]	Primary	Secondary	-	Secondary
	Animal Model Quality Assessment (AMQA)[22]	Primary	-	-	Secondary
	Animal Model Framework (AMF)[26]	-	-	Primary	Secondary
	3Vs Framework (3Vs)[19]	Primary	Secondary	-	-
Mechanism-Centric	Preclinical Assessment for Translation to Humans (PATH) [25]	Secondary	-	-	Primary
	Guidelines On Target Assessment (GOT-IT)[17]	Secondary	-	-	Primary
	Open Targets Platform (OTP)[30]	Secondary	-	-	Primary
Evidence-Centric	Animal Research: Reporting of In Vivo Experiments (ARRIVE) 2.0 [15]	Secondary	Primary	-	-
	PREPARE (Planning Research and Experimental Procedures)[13]	Secondary	Primary	-	-
	Design and Execution of Protocols (DEPART) [14]	Secondary	Primary	-	-
	EQIPD Quality System [18]	Secondary	Primary	-	-
	SYRCLE’s Risk of Bias (RoB) Tool [20]	Secondary	Primary	-	-
	Replacement Checklist [21]	-	-	Secondary	Primary
Regulatory - Influenced	Translatability Score [27, 28]	-	-	Secondary	Primary
	Probability of Pharmacological Success (PoPS)[10]	-	-	Secondary	Primary
	GRADE4Animals [24]	-	Primary	-	Secondary
	Clinical Relevance Assessment of Animal Preclinical Research (RAA) Tool[23]	Primary	-	Secondary	-
	NAMs Scientific Confidence Framework (NAMs-SCF)[8]	Primary	Secondary	-	-
	Decision-Theoretic Framework (D-TF)[31]	-	-	Primary	Secondary

 The four cross-cutting dimensions (i) construct/external validity, (ii) internal validity/risk of bias, (iii) predictive validity/performance, and (iv) go/no-go and portfolio progression, should be read as operational phases. In addition, while most of the tools touch multiple dimensions, they appear to be optimized for one recurring decision need.

Model-centric instruments treat model choice as the dominant determinant of translatability and therefore prioritize construct/external validity. FIMD is primary here via structured comparison across disease-relevance domains, with secondary value in exposing evidence gaps that affect internal validity [12, 27]. AMQA similarly interrogates human-condition alignment and model concordance, but is less oriented to predictive-performance outputs [22]. AMF most directly targets predictive validity by explicitly comparing animal and human evidence to quantify predictive capacity; however, its portfolio role remains secondary because predictive metrics still require translation into thresholds and benefit-risk/economic framing [26]. The 3Vs framework primarily makes validity assumptions explicit and ordered (primary construct/external; secondary internal), without quantifying predictive performance or delivering a go/no-go algorithm [19].

Mechanism-centric instruments emphasize integrating heterogeneous mechanistic evidence into program logic and milestone decisions. PATH [25], GOT-IT [17], and OTP [30] are therefore primary for go/no-go and portfolio progression and secondary for construct/external validity. PATH decomposes mechanistic links and converts evidence into an auditable decision narrative with explicit gap identification [25]. GOT-IT structures “red flags” and critical-path experiments, emphasizing task sequencing and decision points across academia-industry workflows [17]. OTP prioritizes target-disease association and guides investment prioritization; its practical function is progression governance rather than study-conduct certification [30].

Evidence-centric instruments predominantly mitigate internal validity threats and improve reproducibility, transparency and synthesis readiness. ARRIVE 2.0 [15], PREPARE [13], DEPART [14], EQIPD [18], and SYRCLE RoB [20] are primary for internal validity/risk-of-bias reduction despite different scopes (reporting, planning, execution/analysis guidance, quality systems, bias appraisal). Their predictive-validity role is intentionally limited: they stabilize the methodological substrate on which predictive claims must rest. The Replacement Checklist is grouped here as checklist-driven, but functions primarily as an ethical/governance gate (primary go/no-go; secondary predictive validity insofar as replacement can indirectly improve human relevance) [21].

Regulatory influenced instruments are activated when evidence must be converted into readiness claims for external stakeholders or into explicit progression thresholds. Translatability Score [28, 29] and PoPS [10] are primary for go/no-go because they standardize how multi-source evidence, uncertainty, and “stopper” conditions are compressed into committee-usable metrics. GRADE4Animals is secondary for portfolio decisions by qualifying certainty of the evidence base rather than predicting clinical success [24]. D-TF is the most explicitly portfolio-facing quantitative tool: it translates predictive validity into economic consequences (by calculating: Positive Predictive Value / Net Present Value), making it primary for predictive validity/performance with a material secondary role in portfolio progression by informing where validation investments are rational [31].

Despite their difference in epistemic focus and utility (Table 3), the instruments repeatedly present the same core questions on five themes, suggesting that translational evaluation is reduced to a common decision logic: (1) the model must resemble the human condition at phenotype and mechanism levels; (2) therapeutic response must be anchored by reference drug behaviour; (3) measured outcomes must map onto clinically meaningful endpoints/biomarkers; and (4) evidence must be generated under bias-minimising conditions. Hence these core areas represent the minimum requirements for translational claims by the tool and framework development experts. Importantly we also observed a bifurcated ecosystem: one instrument cluster optimises “decision-grade evidence production” (rigour, reproducibility, reporting), while the other optimises “decision-grade evidence integration” (comparability, prioritisation, and progression).

Overall, our analysis reinforces that “translation” is neither a single concept nor a single tool category. An ideal orchestrating layer should require: (i) model credibility (construct/external validity), (ii) methodological integrity (internal validity and auditability), (iii) mechanism-based integration into milestone logic, and (iv) a readiness-based approach that translates evidence into predefined decision criteria and progression decisions. This taxonomy therefore supports a modular operating model in which tools are deployed as a staged stack (not substitutes), and outputs are tailored to the decision owner (scientific team, ethics committee, portfolio governance, regulator-facing dossier).

## The challenge: orchestration as a limiting step in translational decision-making

As assessment instruments’ availability has expanded, a key bottleneck has shifted from inventing additional ones to operationalising existing ones as an end-to-end, uncertainty-aware E2D workflow. We then analysed the strengths and weaknesses of the translational frameworks. On the strength side, frameworks such as FIMD and AMQA represent a meaningful advance over narrative model selection by forcing explicit documentation of biological discordance and translating expert intuition into semi-quantitative, auditable outputs. PATH further strengthens the evidentiary posture by dissecting therapeutic hypotheses into a mechanistic chain, identifying where assumptions replace data and thereby elevating the quality of Investigator’s Brochure-style rationales. Across the tool landscape, there is also a clear convergence on “core” translational prerequisites, suggesting that the tool developers’ community broadly agrees on what must be asked before human exposure is justified. Importantly, outputs are intentionally designed for cross-stakeholder interpretability (radar plots, matrices, certainty tiers, annotated diagrams), which improves organisational alignment and creates governance-ready audit trails. Finally, various checklists re-position ethics review from passive assurance to active verification by requiring a documented justification for rejecting non-animal new approach methodologies (NAMs), thereby operationalising Replacement [32] as a measurable process rather than a rhetorical commitment.

On the other side, we observed that weaknesses are systemic, more than tool specific. A recurrent limitation is that many instruments operationalise plausibility of human relevance/biological fidelity (phenotype, orthology, histology, and mechanistic concordance) without demonstrating that such fidelity translates into improved positive predictive value for clinical outcomes. As said, this is not a flaw of any single tool; it reflects the retrospective bias, because clinical outcomes are often unavailable and pharmacological validation in model-centric tools often depends on published literature, where negative results are structurally suppressed, inflating the real performance [33]. This is a major limitation that creates an open-loop nature of translational assessment. While prospective evaluation has been reported in one setting [34], prospective validation remains limited across the assessment instruments landscape and is rarely standardised across disease areas or decision contexts. Even if all considered instruments appear proficient at structuring experts’ judgements and governing decision-making, they are underpowered in predicting clinical translatability.

Several instruments transform judgments into precise scores, and without explicit uncertainty representation and validation. Such scores risk false precision and can create a misleading impression of a straightforward line of reasoning that is complex and difficult to scientifically justify. Quantification can still be valuable when it is explicitly treated as a decision aid rather than a measurement, and when uncertainty and validation status are surfaced alongside point estimates. A practical safeguard is to require every composite score to be accompanied by (i) its dominant assumptions, (ii) a confidence/indirectness profile, and (iii) explicit breach conditions that override aggregation.

The translational assessment instruments ecosystem also exhibits a key orchestration gap. Two illustrative scenarios show how continuity can be lost when assessment instruments are used as standalone. First, model-selection tools may identify a model as fit for a specified purpose while documenting weak pharmacological validation, endpoint discordance, biological differences, or missing evidence in particular domains. If only the final model choice or summary rating is transferred, downstream teams may interpret the model as generally qualified rather than conditionally suitable for a defined inference. Second, risk-of-bias and certainty-assessment instruments may identify bias, imprecision, indirectness, or incomplete reporting. If these findings are not linked to later translational synthesis, the contributing efficacy evidence may be incorporated without an appropriate reduction in confidence, and a downstream score or probability estimate may appear more certain than the evidence supports. The continuity problem therefore concerns not only retention of data, but also retention of the conditions governing how those data should be interpreted, weighted, and updated. In this view, there is no workflow that preserves decision intent, assumptions, and uncertainty across the discovery-to-FIH continuum. At minimum, each workflow output should carry: (i) claim identification; (ii) source set; (iii) uncertainty tags (indirectness, bias, imprecision); (iv) decision owner; (v) breach/veto conditions; (vi) update triggers. This is not a minor operational inconvenience; it is a core barrier because it prevents the accumulation of evidence into a continuous “golden thread”, forcing repeated manual synthesis and enabling validity signals to be lost at handoffs.

Moreover, translational assessment remains fragmented across efficacy and safety. The safety evaluation can be anchored in quantitative predictive-performance frameworks, whereas efficacy evaluation often relies on disease-model mechanistic relevance in construct or face validity. These evidence streams should be integrated through a metric that balances efficacy and safety across contexts. The acceptable trade-off depends on disease severity, unmet needs, available alternatives, intended population, regional or regulatory expectations, and stakeholder values. Future evidence-to-decision workflows should therefore make efficacy–safety assumptions, safety margins, uncertainty, and breach conditions explicit for a defined context of use, while leaving final weighting and progression thresholds to accountable decision-makers.

An interoperable E2D workflow may be useful as an enabling layer for retrieval-grounded extraction and normalisation, but it does not remove the need for explicit decision rules, validation, and governance.

In principle, Bayesian or decision-theoretic framing can express uncertainty transparently and enable calibrated updating, but only when priors, endpoints and update rules are prespecified, and when outcome-linked datasets are governed, representative and auditable. In most current settings, the binding constraint is not mathematics but the availability of suitable outcome-linked data and a validation plan [10, 31]. Replacing static scores with dynamic Bayesian updating would allow organisations to treat assays, models, and mechanistic claims as diagnostic tests whose predictive value is estimated empirically and updated over time, enabling Go/No-Go thresholds to be calibrated to measured performance. Importantly, integrating quantitative safety margins into efficacy-focused assessments would increase clinical relevancy earlier. The key enabling condition for these advances is also highlighted in the SWOT analysis (Table 4): global harmonisation through “safe harbour” preclinical and clinical data sharing, including negative results and NAMs performance data, are required to establish base-rate datasets that can calibrate uncertainty and qualify tools statistically. Although more than 560,000 trials are registered on ClinicalTrials.gov, fewer publicly report results [35]. This limits the availability of outcome-linked corpora needed to benchmark and recalibrate translational tools across disease areas and decision contexts.

**Table 4:** SWOT analysis of translational assessment instruments for preclinical-to-clinical decision-making. This table synthesises the strategic strengths, weaknesses, opportunities and threats (SWOT) associated with translational validity assessment. It captures both methodological attributes and operational considerations that shape real-world adoption and decision impact. The “hand-waving’s concept” in the Investigator’s Brochure refers to the practice of using narrative storytelling to bridge logical gaps where hard evidence is missing. Instead of proving every step of the mechanism, the authors rely on persuasive writing to imply connections that have not been tested [31].

	Theme / Issue	Description
Strengths	Structured decision-making	They convert subjective expert judgments into standardised semi-quantitative outputs, forcing explicit documentation of biological discordance and producing an auditable rationale beyond narrative reviews.
	Mechanistic logic decomposition	They decompose therapeutic hypotheses into a structured mechanistic chain, highlighting gaps in evidence and reducing “hand-waving” in Investigator’s Brochure-style rationales.
	Transparency	Many require users to document the rationale behind conclusions, creating an audit trail that is valuable for internal reviews and external scrutiny.
	Interpretability across stakeholders	Outputs are designed to be legible to mixed stakeholders (radar plots, matrices, certainty tiers, annotated diagrams), enabling alignment and communication.
	Define core topics	Independently, frameworks converge on critical translational prerequisites reducing the risk that teams overlook fundamental issues.
	Regulatory defence and ethical compliance	The checklist’s workflow generates a compliance record requiring justification for rejecting NAMs, shifting ethics review from assurance to active verification of replacement [41].
Weaknesses	Workload	Multiple tools require substantial manual evidence gathering, synthesis, and documentation, which increases time-to-decision and hampers real-world uptake.
	Human relevance is inferred, not modelled	Most frameworks use proxies for human relevance (phenotype similarity, orthology, expert judgment) instead of quantitative, function-level predictive modelling calibrated to clinical utility.
	Reliance on biased retrospective data	Pharmacological validation depends on published literature; systematic suppression of negative results propagates publication bias into the model “validation” score, inflating perceived predictiveness.
	Inference of predictive validity with biological similarity	Tools reward anatomical/genetic similarity without demonstrating functional predictivity; high biological fidelity (histology/orthology) does not guarantee the positive predictive value to improve clinical translatability.
	Statistical rigor masking uncertainty	Some aggregate ordinal ratings (1–5) into precise totals (e.g., 4.14), implying linearity and precision that mask uncertainty; these are difficult to justify scientifically and are challenged by evidence-methodology traditions.
	Orchestration gap	Individual tools produce useful outputs, but there is a major pitfall in generating a continuous evidence package that supports discovery-to-FIH decision-making process without manual synthesis. Loss of data flow across tools.
	Safety vs. efficacy fragmentation	Safety studies utilise quantitative predictive performance frameworks while efficacy tools rely on construct and face validity. This difference stems from safety studies being conducted in healthy models and efficacy studies in disease modelling systems. Consequently, a unified translatability metric that combines efficacy and safety risk isn’t feasible.
	Absence of closed-loop feedback	Clinical outcomes (success/failure) are too often not available or accessible and, even when available, are not systematically fed back to recalibrate preclinical tool weighting; frameworks remain uncalibrated and cannot learn which signals correlate with clinical success.
Opportunities	AI-driven evidence synthesis	Fine-tuned LLMs can auto-extract and populate validation domains, lowering curation workload and enabling almost real-time decision support instead of retrospective check listing.
	Global harmonisation via “safe harbour” data sharing	Pre-competitive “safe harbour” consortia enabling sharing of negative results and NAM data, creating base-rate datasets needed to calibrate scores and qualify NAMs statistically.
	“Meta-Layer” Orchestration Architecture	Integrating frameworks into a unified orchestrating meta-layer can create a sequential logic chain in which upstream outputs become explicit constraining variables for downstream evidence assessment. This integration prevents key validity signals from being lost during the transition from discovery to preclinical development.
	Transition to Dynamic Bayesian Updates	Replacing static scores with Bayesian priors enables predictive validity to update as new data appear. By treating R&D outputs as diagnostic tests, organisations can set Go/No-Go thresholds using measured assay performance rather than fixed p-values or subjective judgment.
	Context-specific safety into efficacy assessment	Human-relevant exposure and safety-margin information should be displayed alongside efficacy evidence so that decision-makers can judge whether the observed efficacy signal is plausible at clinically acceptable exposures for the intended population, indication, and regulatory context.
Threats	Tool-IP economic paradox	R&D incentives disproportionately reward patentable drug assets over translational decision tools, whose value spills over to competitors, resulting in sustained underinvestment in tool validation and slower maturation of predictive frameworks.
	Regulatory double-standards	Lack of harmonisation forces dual testing (animals for some regions; NAMs for others), pushing companies toward lowest-common-denominator animal testing to preserve global market access, undermining replacement adoption.
	Low (or lack of) adoption	Without workflow integration and automation, complex frameworks risk becoming compliance-driven “box-ticking” completed afterwards for review, rather than shaping study design; perpetuating reliance on low-predictive pre-clinical models.
	False precision of aggregated scores	Aggregating ratings (e.g., colour matrices or 1–5 scales) into a single score can create false precision, hiding uncertainty and sparse evidence.

The analysis of threats underscores the incentive and governance problem, including the methodological one, for achieving such orchestration model (or meta-tool). The Tool-Intellectual Property paradox [31] predicts persistent underinvestment in tool validation unless precompetitive consortia, regulatory qualification pathways, or outcome-linked incentives outweigh disincentives. Similarly, regulatory double standards risk keeping the field into lowest-common-denominator animal testing, undermining Replacement even when superior NAM evidence exists. Finally, without workflow integration, the field risks institutionalising a compliance culture in which frameworks are completed retrospectively for control reasons rather than prospectively to shape experimental design.

## Toward interoperable E2D workflows: an orchestration model for translational evidence integration

Our review suggests that the main problem in translational assessment is not a lack of frameworks, checklists, or scoring tools. The field already has instruments that address model relevance, study quality, reporting, mechanistic reasoning, and program-level decision support. The unsolved problem is how the corresponding outputs are carried forward across the development pathway. In current practice, they are often generated separately, interpreted locally, and then revisited at the next milestone with limited continuity. As a result, the rationale for progression is repeatedly rebuilt rather than accumulated as a traceable body of evidence.

On this basis, we propose an orchestration model that links existing translational assessment instruments into a staged E2D workflow to make their outputs interoperable. In practical terms, this means that evidence generated at one step should remain usable at the next step without losing the assumptions, limitations, and uncertainty that shaped the original judgment. Table 5 summarizes the main workflow steps, the decision value of each step, the expected outputs, and the representative instruments that can inform them. The starting point in such a model is to define the CoU before evidence is assembled. This is important because translational decisions are not all of the same kind. Selecting a disease model, deciding whether a package is mature enough for FIH consideration, and comparing several assets for portfolio prioritization each require different evidence outputs and different levels of confidence. A translational workflow should therefore begin by making explicit what decision is being supported, who owns that decision, what claim is being tested, and which conditions would stop progression even if other findings appear favorable [36].

**Table 5:** Proposed orchestration model for translational evidence integration. The model links existing assessment instruments into a staged evidence-to-decision workflow. It is not presented as a new scoring system or a validated platform. Each step is designed to preserve source traceability, uncertainty, and decision context so that evidence generated at one stage remains usable at the next

Workflow step	Core question	Primary decision value
1. CoU and decision framing	What decision is being supported, for whom, and under what progression constraints?	Prevents generic evidence packages by aligning assessment to a specific use case such as model selection, FIH readiness, or portfolio prioritization.
2. Structured evidence assembly	What evidence supports or weakens the translational claim, and how traceable is it?	Converts isolated tool outputs into reusable evidence assets rather than one-off local judgments.
3. Translational integration	Does the assembled evidence support a specific translational claim across biological relevance, methodological credibility, pharmacological plausibility, and clinical interpretability?	Moves the workflow from tool completion to claim-level judgment.
4. Deliberate challenge	What findings, analogues, or alternative explanations would weaken the case for progression?	Reduces overconfidence by making disconfirming evidence, fail-alike, endpoint mismatch, and safety constraints visible before governance review.
5. Decision-facing synthesis at governance gate	Given the current evidence and uncertainty, should the program advance, pause, or stop for this context of use?	Converts scientific synthesis into accountable governance artefacts without masking uncertainty.
6. Managed updating and validation	How should new preclinical or clinical information change the assessment over time?	Preserves continuity across handoffs while avoiding the assumption of automatic self-learning.

The next step is structured evidence assembly. Here, the reviewed instruments keep their original role, but their outputs are captured in a shared decision-ready format. At minimum, each output should specify the translational claim being addressed, the underlying evidence sources, the main sources of uncertainty, and the conditions under which the conclusion would need to be revised. This shifts the output from a one-off local assessment to a reusable evidence asset. A model-selection exercise, for example, should not end with a simple statement that one model is preferable. It should document why that model was considered fit for purpose, what biological gaps remain, and which downstream inferences depend on those gaps being acceptable.

A third step is translational integration. At this stage, the question is no longer whether an individual framework has been completed appropriately, but whether the assembled evidence supports a specific translational claim. Based on the tools identified in this review, four domains appear to require integration: biological relevance, methodological credibility, pharmacological plausibility, and clinical interpretability. Biological relevance addresses whether the model reflects aspects of the human condition that matter for inference. Methodological credibility addresses whether the evidence is sufficiently robust to support decision-making. Pharmacological plausibility addresses whether the observed effects occur within an exposure range that could be meaningful in humans and remain compatible with safety constraints. Clinical interpretability addresses whether the endpoints, biomarkers, or mechanistic readouts can inform a human development decision rather than only a preclinical narrative. Considered together, these domains offer a more realistic basis for progression than a single summary score.

A useful feature of this model is the explicit separation between evidence-facing outputs and decision-facing outputs. Evidence-facing outputs are designed for scientific scrutiny. They retain detail, source traceability, and uncertainty. Decision-facing outputs serve a different purpose. They are intended for portfolio committees, translational governance groups, ethics bodies, and regulators who need concise and accountable progression documents. These outputs should state what is supported, what remains uncertain, what would block progression, and what new information would require reassessment. Making this distinction explicit reduces a common problem in translational development, where a narrative scientific summary is treated as an implicit recommendation even though decision thresholds were never clearly defined [36–38]. 

The model should also include a deliberate challenge function. Translational assessment is more credible when it actively searches for disconfirming evidence rather than only organizing supportive findings. In practical terms, this means looking for negative studies, historical fail-alikes, inconsistent model behavior, weak pharmacological anchoring, endpoint mismatch, and safety constraints that would make an efficacy signal clinically irrelevant. This step is especially important in areas where publication bias and selective reporting may distort the apparent strength of the evidence base [39, 40]. 

Within such a workflow, computational support may have a useful but bounded role. Its most defensible contribution is at the level of evidence management: retrieving traceable sources, extracting prespecified fields, normalizing terminology, and maintaining versioned records across workflow stages. That could lower the curation burden that currently limits the prospective use of many translational tools. It should not be confused with automated progression decisions. Decisions about advancement, stopping rules, and recalibration remain governance functions and require accountable human judgment. Any attempt to update the workflow using downstream clinical outcomes should therefore be treated as a formal validation program rather than an assumed feature.

This proposal is intended as a conceptual model derived from the present review, not as a validated platform. Its main value is to provide a structured way to connect existing translational assessment tools across development stages while preserving traceability and uncertainty. Whether such an approach improves predictive performance, reduces duplication of effort, or supports more robust first-in-human decisions will require prospective evaluation.

## Limitations of this review

This review has several limitations. First, it was designed as a scoping review to map the published ecosystem of translational assessment instruments rather than as a systematic review intended to estimate effect sizes or formally compare predictive performance. Second, the search was restricted to English-language peer-reviewed journal articles indexed in PubMed and supplemented by citation snowballing; this approach may under-represent internal industry tools, non-English instruments, and frameworks described outside PubMed-indexed literature. Third, because the field uses heterogeneous terms and overlapping concepts, classification of instruments by dominant conceptual focus necessarily involved interpretive judgment, although screening and extraction were performed by two reviewers and disagreements were resolved by discussion. Fourth, the published literature provides uneven evidence about real-world uptake, prospective validation, and benchmarking against downstream human outcomes. Fifth, this review did not directly observe organisational handoffs, quantify the frequency of continuity loss, or compare integrated and non-integrated workflows. The scenarios described therefore represent plausible failure modes inferred from the functions and outputs of the mapped instruments. Similarly, the proposed benefits of AI-assisted evidence capture and retrieval remain conceptual and would require evaluation for extraction accuracy, retrieval completeness, reproducibility, auditability and impact on decision quality. Finally, the interoperability framework proposed here is an author-derived conceptual synthesis based on the reviewed literature and should not be interpreted as a validated decision-support system.

## Conclusions

Structured translational assessment instruments already cover important parts of the preclinical-to-FIH pathway, particularly model relevance, methodological rigor, reporting quality, and mechanistic organization of translational claims. What remains underdeveloped is not simply another tool, but a way to preserve assumptions, uncertainty, and decision intent as evidence moves across discovery, preclinical development, and FIH governance.

This review suggests that the current landscape is stronger in decision-grade evidence production than in decision-grade evidence integration. We therefore propose a conceptual interoperability framework in which evidence-facing outputs remain traceable and uncertainty-aware, while decision-facing outputs are released only within explicit governance rules. Such a framework should clearly define the CoU of each instrument, ensuring that the resulting outputs are appropriate for the specific decision-making scenario being addressed. The value of such workflow now needs to be tested prospectively against downstream human outcomes, decision auditability, and duplication of curation effort.

## Supplementary Information

Below is the link to the electronic supplementary material.

Supplementary Material 1

## Data Availability

All data generated or analysed during this study are included in this published article and its supplementary information files. The extraction framework and instrument-level comparative data are available from the corresponding author on reasonable request.

## Abbreviations

AI: Artificial intelligence
AMF: Animal Model Framework
AMQA: Animal Model Quality Assessment
ARRIVE: Animal Research: Reporting of In Vivo Experiments
CoU: Context of use
D-TF: Decision-Theoretic Framework
DEPART: Design and Execution of Protocols
EQIPD: European Quality in Preclinical Data
E2D: Evidence-to-Decision
FIH: First-in-human
FIMD: Framework to Identify Models of Disease
GOT-IT: Guidelines On Target Assessment
LLM: Large language model
NAMs: New approach methodologies
NAMs-SCF: New Approach Methodologies Scientific Confidence Framework
OTP: Open Targets Platform
PATH: Preclinical Assessment for Translation to Humans
PoPS: Probability of Pharmacological Success
PRISMA-ScR: Preferred Reporting Items for Systematic Reviews and Meta-Analyses extension for Scoping Reviews
RAA: Clinical Relevance Assessment of Animal Preclinical Research
RoB: Risk of bias
