# Correction to: Translational assessment instruments for preclinical to first-in-human decision-making: a scoping review

**DOI:** 10.1186/s12967-026-09140-8

**Published:** 2026-11-12

**Authors:** Marco Straccia, David Mawufemor Azilagbetor, Celean Camp, Guilherme S. Ferreira, Maria Laura Garcia-Bermejo, Annalisa Gastaldello, Kurinchi Gurusamy, Francesco Pappalardo, Merel J. Ritskes-Hoitinga, Fabrizio Rossi, Danilo Tagle, Martin Wehling, Francesca Pistollato

**Affiliations:** 1 FRESCI by Science&Strategy SL, Barcelona, Spain; 2 Institute for Biomedical Ethics, University of Basel, Basel, Switzerland; 3 Replacing Animal Research, Nottingham, UK; 4 Utrecht University, Utrecht, The Netherlands; 5 European Infrastructure for Translational Medicine (EATRIS), Amsterdam, The Netherlands; 6 Ramón y Cajal Health Research Institute (IRYCIS), Madrid, Spain; 7 European Commission, Joint Research Centre (JRC), Ispra, Italy; 8 Division of Surgery and Interventional Science, University College London (UCL), London, UK; 9 Department of Drug and Health Sciences, University of Catania, Catania, Italy; 10 Aarhus University, Aarhus, Denmark; 11 Office of Special Initiatives, National Center for Advancing Translational Sciences (NCATS), National Institutes of Health, Bethesda, MD USA; 12 Clinical Pharmacology, Heidelberg University, Mannheim, Germany; 13 Humane World for Animals International, Brussels, Belgium

**Correction to: Journal of Translational Medicine (2026) 24:1266**

**https://doi.org/10.1186/s12967-026-08604-1**

In this article an incomplete figure appeared as Fig. 1.; the figure should have appeared as shown below. The original online version of this article was revised.

**Fig. 1:**
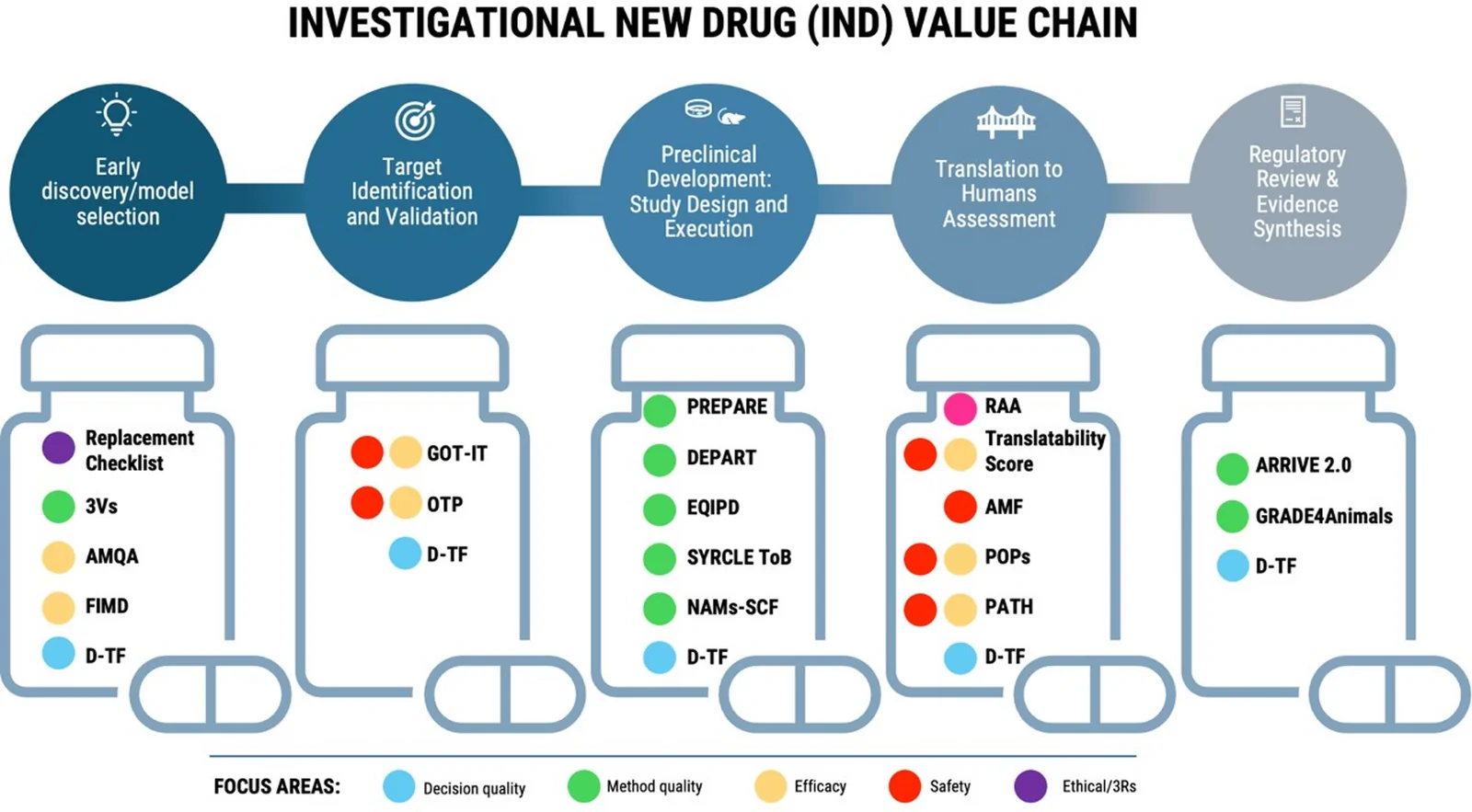
Where translational instruments sit in the discovery-to-FIH workflow. Instruments are positioned by their most common point of activation; many span multiple stages. Colour labels indicate the dominant focus of the instrument’s output (decision quality, method quality, efficacy, safety, ethical/3Rs). (1) Early Discovery / Model Selection: The foundational stage where researchers identify, evaluate, and select the most appropriate experimental systems to serve as surrogates for human biology and disease. The goal is to establish Construct Validity and Face Validity by ensuring the chosen model accurately reflects the human condition’s aetiology, pathophysiology, and symptomatology. Selecting a poorly predictive model at this stage is a primary driver of downstream translational failure. (2) Target Identification and Validation: The process of discovering a novel molecular entity (such as an enzyme or receptor) linked to a disease process, and generating rigorous evidence that pharmacological modulation of this target will yield a therapeutic benefit in a defined patient population. Before designing molecules, researchers must confirm that the target is causally linked to the disease, “druggable,” and lacks severe safety liabilities (on-target toxicity). It also involves evaluating the competitive landscape and intellectual property viability. (3) Preclinical Development: Study Design and Execution: The operational phase where experiments on the selected models are planned, conducted, and documented to generate robust data on a therapeutic candidate’s efficacy and safety prior to human testing. The primary focus here is maximizing Internal Validity by minimizing systematic errors and biases. This requires meticulous attention to experimental hygiene, including proper randomization, blinding, sample size calculation, and transparent reporting. (4) Translation to Humans Assessment: The synthesis and evaluation of accumulated preclinical pharmacological, toxicological, and mechanistic data to predict clinical efficacy and safety, directly informing the critical “Go/No-Go” decision for first-in-human (Phase I) trials. This stage translates dynamic responses from the model to the patient. It requires estimating the probability of success by integrating uncertainties around drug potency, exposure, safety margins, and the relevance of biomarkers. (5) Regulatory Review & Evidence Synthesis: The formal, systemic aggregation and critical appraisal of a body of preclinical evidence to support high-stakes decisions, such as Investigational New Drug (IND) approvals by regulatory agencies (FDA/EMA), clinical trial authorizations, or ethical committee harm-benefit analyses. This phase relies on systematic reviews and meta-analyses to determine the aggregate certainty, robustness, and clinical relevance of the data. It acts as the final gatekeeper before exposing human subjects to novel interventions

