# Supplementary material for: Translational assessment instruments for preclinical to first-in-human decision-making: a scoping review
Source: J Transl Med. 2026 Jul 13;24:1266. doi: 10.1186/s12967-026-08604-1 (PMC13647670; doi:10.1186/s12967-026-08604-1)
Supplement: Supplementary file 1 [file 12967_2026_8604_MOESM1_ESM.docx]

# Supplementary Information

**Translational assessment instruments for preclinical to first-in-human decision-making: A scoping review**

**Authors:** Marco Straccia^1^, David Mawufemor Azilagbetor^2^, Celean Camp^3^, Guilherme S Ferreira^4^, Maria Laura Garcia-Bermejo^5,6^, Annalisa Gastaldello^7^, Kurinchi Gurusamy^8^, Francesco Pappalardo^9^, Merel J. Ritskes-Hoitinga^4,10^, Fabrizio Rossi^1^, Danilo Tagle^11^, Martin Wehling^12^, and Francesca Pistollato^13^

**Affiliations:**

1. FRESCI by Science&Strategy SL, Barcelona, Spain
2. Institute for Biomedical Ethics, University of Basel, Basel, Switzerland
3. Replacing Animal Research, Nottingham, UK
4. Utrecht University, Utrecht, the Netherlands
5. European Infrastructure for Translational Medicine (EATRIS), Amsterdam, the Netherlands
6. Ramón y Cajal Health Research Institute (IRYCIS), Madrid, Spain
7. European Commission, Joint Research Centre (JRC), Ispra, Italy
8. Division of Surgery and Interventional Science, University College London (UCL), London, UK
9. Department of Drug and Health Sciences, University of Catania, Catania, Italy
10. Aarhus University, Aarhus, Denmark
11. Office of Special Initiatives, National Center for Advancing Translational Sciences (NCATS), National Institutes of Health, Bethesda, MD, USA
12. Clinical Pharmacology, Heidelberg University, Mannheim, Germany
13. Humane World for Animals International, Brussels, Belgium

**Corresponding author:**

Dr. Marco Straccia, PhD MBA

E-Mail: [marco.straccia@fre-sci.com](mailto:marco.straccia@fre-sci.com)

ORCID: 0000-0003-3903-4163

Supplementary Table 1. Authors’ definitions of key concepts related to evidence assessment and decision-making in the drug development pipeline.

| **Concept** | **Meaning** | **Decision** | **Timeline** |
| --- | --- | --- | --- |
| **Preclinical evidence** | Data generated during discovery and preclinical development, including in vitro, in vivo, pharmacokinetic, pharmacodynamic, and toxicology studies used to assess a drug candidate's potential. | What evidence supports further development of the candidate? | Generated throughout discovery and preclinical development. |
| **Preclinical-to-clinical translation decisions** | Decisions regarding whether and how preclinical findings can inform clinical development, including clinical trial design, dosing, safety assessment, and patient selection. | Are the preclinical findings sufficiently relevant and predictive to support clinical development? | Primarily during late preclinical development and throughout clinical development. |
| **Preclinical-to-human decision-making** | The process of evaluating preclinical evidence to determine whether a drug candidate is sufficiently supported to enter human testing. | Does the available evidence justify initiating studies in humans, and under what conditions? | Occurs during the transition from preclinical development to first-in-human studies. |
| **First-in-human (FIH) decision** | The decision to administer a drug candidate to humans for the first time, based on an assessment of preclinical safety, pharmacology, and pharmacokinetic data. | Is the candidate sufficiently safe and supported to begin human testing? | Final decision point before initiation of Phase I or equivalent studies. |
| **Portfolio progression** | Advancement of a drug candidate through successive stages of the drug development pipeline based on accumulated evidence. | Should the candidate advance, be prioritised, paused, or terminated? | Occurs throughout the entire drug development lifecycle. |
| **Go/no-go algorithm** | A predefined set of criteria used to support progression decisions at key development milestones. | Should the candidate proceed to the next stage, be modified, or be discontinued? | Applied at predefined decision points across development. |
| **Decision-support instruments** | Tools, frameworks, methodologies, or AI-based systems used to generate, organise, or evaluate evidence for decision-making. | What evidence supports a given development decision? | Can be used throughout the drug development pipeline. |

# Supplementary Information on review methods and evidence base

We conducted a scoping literature search informed by PRISMA Scoping Review principles[1] to identify peer-reviewed publications presenting structured assessment instruments (tools, frameworks, checklists, scoring systems or algorithms) explicitly designed to inform preclinical to FIH translation decisions. The search strategy targeted three concept blocks: (i) preclinical models, (ii) predictive validity and/or clinical translatability, and (iii) assessments/decision-support instruments. We used PubMed as the primary database and complemented retrieval by citation snowballing to refine coverage[2]. Given the aim of mapping and classifying instruments, and not estimating effect sizes, we report our screening and selection process transparently, but do not present this work as a full systematic review.

## Eligibility and exclusion criteria

We included English-language peer-reviewed journal articles indexed in PubMed from 1 January 2006 to 1 December 2025. This scope favours instruments with biomedical journal visibility and may under-represent internal industry frameworks, non-English tools and instruments described outside PubMed-indexed journals; we treat the mapping as illustrative of the published ecosystem rather than exhaustive. We focused on a time period marked by expansion of translational science initiatives as the launch of the NIH Clinical and Translational Science Awards (CTSA)[3], the establishment of the Predictive Safety Testing Consortium (PSTC)[4], the Biomarkers Consortium[5], and the publication of foundational meta-analyses quantifying the translational gap[6]. We included peer-reviewed journal articles that proposed, validated or applied a structured assessment instrument (tool, framework, checklist, scoring system or algorithm) intended to inform preclinical-to-clinical translation decisions in therapeutic development. Instruments could target in vivo, in vitro, in silico or NAM-based evidence, provided their stated purpose related to informing translation to humans. We excluded articles describing instruments intended exclusively for human clinical trials without explicit adaptation for preclinical evidence, and instruments narrowly disease-specific without an explicit claim of transferability across programmes. We also excluded regulatory chemical hazard/risk assessment frameworks from the primary mapping because their unit of analysis and decision context differ materially from therapeutic efficacy translation.

## Study selection and screening

Records retrieved from PubMed and citation snowballing were deduplicated and screened for eligibility by two reviewers (M.S. and F.R.) using a web-based screening workflow[7]. Disagreements at title/abstract or full-text stage were resolved by discussion, with explicit confirmation that each included record described a distinct assessment instrument intended to inform preclinical-to-clinical translation decisions.

## Data Extraction and Analysis

For each included study, M.S. and F.R. extracted a qualitative set of variables to support comparative synthesis of translatability assessment approaches. Extracted fields were (i) tool/framework identity, (ii) intended use case, (iii) assessment domains covered, (iv) implementation requirements, and (v) validation status. We then conducted a narrative synthesis supported by conceptual mapping. Tools and frameworks were grouped into families based on conceptual focus and/or primary intended utility. We summarised domain coverage, decision contexts, degree of operationalisation, and evidence of validation or applied use, and we described recurring assessment domains and validation approaches across the included literature.

## PubMed search query

The following PubMed query, using Medical Subject Headings (MeSH) and keyterms, retrieved 1724 results: (("models, animal"[MeSH Terms] OR "drug evaluation, preclinical"[MeSH Terms] OR "animal model*"[Title/Abstract] OR "preclinical study"[Title/Abstract] OR "preclinical research"[Title/Abstract] OR "non-clinical"[Title/Abstract] OR "in vivo"[Title/Abstract] OR "animal experimentation"[Title/Abstract]) AND ("translational research, biomedical"[MeSH Terms] OR "Reproducibility of Results"[MeSH Terms] OR "translational value"[Title/Abstract] OR "clinical relevance"[Title/Abstract] OR "predictive validity"[Title/Abstract] OR "translatability"[Title/Abstract] OR "external validity"[Title/Abstract] OR "construct validity"[Title/Abstract] OR "internal validity"[Title/Abstract] OR "predictive capacity"[Title/Abstract]) AND ("framework"[Title/Abstract] OR "assessment tool"[Title/Abstract] OR "checklist"[Title/Abstract] OR "guideline*"[Title/Abstract] OR "scoring system"[Title/Abstract] OR "quality assessment"[Title/Abstract] OR "risk of bias"[Title/Abstract] OR "decision support"[Title/Abstract] OR "validation tool"[Title/Abstract] OR "meta-tool"[Title/Abstract])) AND ((2006/1/1:2025/12/1[pdat]) AND (english[Filter])).

Supplementary Table 2

| **Concept block** | **Search Key** |
| --- | --- |
| Preclinical/animal models | **MeSH:** "models, animal"; "drug evaluation, preclinical" **Title/Abstract:** "animal model*"; "preclinical study"; "preclinical research"; "non-clinical"; "in vivo"; "animal experimentation" |
| Translational value / validity / reproducibility | **MeSH:** "translational research, biomedical"; "Reproducibility of Results" **Title/Abstract:** "translational value"; "clinical relevance"; "predictive validity"; "translatability"; "external validity"; "construct validity"; "internal validity"; "predictive capacity" |
| Frameworks / tools / quality systems | **Title/Abstract:** "framework"; "assessment tool"; "checklist"; "guideline*"; "scoring system"; "quality assessment"; "risk of bias"; "decision support"; "validation tool"; "meta-tool" |

Supplementary Material Figure 1: PRISMA Flowchart of the reviewing process.

Supplementary Table 3: Instrument-level map of translational assessment tools, detailed version.

| Instruments | Utility | Step 1: Inputs | Step 2: Process | Step 3: Outputs Type |
| --- | --- | --- | --- | --- |
| **2009 - Animal Model Framework (AMF)**[8] | Aligning models to pipeline phases; removing models with poor predictive capacity. | Retrospective non-clinical data and Phase I clinical adverse event data. | Drugs mapping Animal vs Human. | QUANTITATIVE: Sensitivity %, Specificity %, Predictive Capacity % |
| **2009 (2017) - Translatability Score**[9,10] | Portfolio risk assessment; Go/No-Go decisions; guiding biomarker and early clinical trial development | Project data: in vitro/in vivo, human genetics, biomarker availability, clinical need, clinical trials, IP status (plus any relevant clinical data) | Rate items 1 (weak) to 5 (strong); apply predefined weights (customisable by disease area); aggregate into a sum score; includes “stopper” logic | QUANTITATIVE: Numerical risk/translatability score (e.g., 4.14; >4 = fair-to-good); explicit stopper identification (project “untranslatable” if critical factor is 0) |
| **2014 - SYRCLE’s Risk of Bias (RoB) Tool**[11] | Assessing internal validity; weighting evidence reliability. | Methodological details: sequence generation, allocation concealment, housing, blinding, outcome. | The reviewer answers signalling questions across 6 domains of bias: selection, performance, detection, attrition, reporting, others. | QUALITATIVE: Answers are Yes to indicate a low risk of bias; No for a high risk of bias; and Unclear when insufficient details reported in the study. |
| **2017 - DEPART (Design and Execution of Protocols)**[12] | Improving experimental rigor and reporting to ensure studies can be included in systematic reviews. | Study objectives; model choice rationale; reagents; blinding/randomisation plans; statistical analysis plans. | Users consult checklist items at 3 distinct stages: Before (Design), During (Conduct), and After (Analysis) the experiment. | QUALITATIVE: Checklist compliance ensuring methodological rigor and minimising bias. |
| **2018 - GRADE4Animals**[13] | Decisions on proceeding to clinical trials; regulatory decisions on exposure limits; utility of further animal studies. | Systematic review data, effect estimates, risk of bias assessments, indirectness factors | Start with the assumption of High Quality (for randomised experiments). Downgrade based on 5 factors: risk of bias; imprecision; inconsistency, publication bias; indirectness. | SEMI-QUANTITATIVE: Certainty rating as High, Moderate, Low, Very Low |
| **2018 - PREPARE (Planning Research and Experimental Procedures)**[14] | Improving quality, reproducibility, and safety of animal studies before experiments begin (Planning phase). | Study hypothesis; literature search results; legal/ethical constraints; details on facility and division of labour. | Researchers run through a checklist of 15 topics covering Formulation of the study, Dialogue with facility, and Quality Control. | QUALITATIVE: A completed checklist acting as an action plan for study execution and collaboration. |
| **2019 - Framework to Identify Models of Disease (FIMD)**[15] | Selecting the most relevant animal model to test drug efficacy; support for Institutional Review Board (IRB) approval. | Scoping review using MeSH/Emtree to collect evidence on model + human indication; domain evidence on epidemiology, symptoms, genetics, biochemistry, histology, pharmacology (response to known drugs) | User answers questions across 8 domains (epidemiology; symptomatology and natural history; genetics; biochemistry; aetiology; histology; pharmacology; endpoints); weighted scoring totals 0-100; includes pharmacological validation (effective human drugs effective in model; ineffective drugs fail) | QUANTITATIVE: Radar plot across 8 axes; Validation level (e.g., moderately validated); Uncertainty factor for missing data |
| **2020 - Animal Research: Reporting of In Vivo Experiments (ARRIVE) 2.0**[14] | Improving transparency, reproducibility, and reliability of animal research findings; allowing readers to scrutinise methodological rigour. | Manuscript details regarding: The "Essential 10" (e.g., Study Design, Sample Size, Inclusion/Exclusion criteria, Randomisation, Blinding, Outcome measures) and the "Recommended Set" (e.g., Abstract, Background, Protocol registration). | Checklist Compliance: Authors or reviewers cross-reference the manuscript against specific items to ensure minimum reporting requirements are met. The Essential 10 constitute the minimum requirement, while the Recommended Set adds context. | QUALITATIVE: A completed checklist ensuring transparent and accurate reporting; a manuscript containing sufficient detail to allow replication |
| **2021 - Animal Model Quality Assessment (AMQA)**[16] | Informing model selection; supporting Harm-Benefit Analysis (HBA) for ethical review | Team-collected internal/external data (investigators, vets, pathologists) on target biology and proposed model | Primary + secondary questions across 5 areas (human condition; model physiology; model pharmacology; model disease; reproducibility); assigns Red/Amber/Green based on concordance | SEMI-QUANTITATIVE: Colour matrix plus narrative justification documenting weaknesses and mitigations |
| **2021 - Clinical Relevance Assessment of Animal Preclinical Research (RAA) Tool**[17] | Determining if therapeutic findings are likely to translate to human management; "Fund/Approve" vs "Reject". | Study design details (blinding, randomisation), bias controls, and reproducibility data. | Evaluate 8 domains using questions: construct validity, experimental design;  bias; external validity; reproducibility; study conclusions; research integrity and  transparency. | SEMI-QUANTITATIVE: Domain concerns: Low/Mod/High; Overall: High/Uncertain relevance. |
| **2021 - Guidelines On Target Assessment (GOT-IT)**[18] | Prioritising target assessment activities; identifying "red flags"; facilitating academia-industry collaboration. | Project data relevant to 5 Assessment Blocks: Linkage, Safety, Microbial targets, Strategic issues, technical feasibility. | Users answer Critical Path Questions (CPQs) to select relevant blocks, then Experimental Approach Questions (EAQs) to define tasks, followed by Data Quality checks. | QUALITATIVE: A project-specific critical path; identification of "Go/No-Go" decision points and milestones; detection of gaps. |
| **2021 - Open Targets Platform (OTP)**[19] | Systematic target identification and prioritisation based on integrated genomic and phenotypic evidence. | 20+ data sources including Genetics, Somatic mutations, Drugs, Pathways, RNA expression, Text mining, and Animal models. | Data integration followed by evidence scoring (0-1); calculation of harmonic sums for data types; prioritisation based on attributes like tractability and safety. | QUANTITATIVE: An "Overall Association Score" ranking targets for a specific disease; specific tractability and safety assessments. |
| **2021 - EQIPD Quality System**[20] | Boosting innovation and ensuring data are "fit for intended use" in non-regulated preclinical research. | Research unit processes; data integrity measures; study plans; governance structures. | Implementation of 18 core requirements (e.g., transparency, traceability) followed by self-assessment or external assessment. | QUALITATIVE: Quality System certification; declaration that data are generated under a rigour-enhancing framework. |
| **2021 - 3Vs Framework (3Vs)**[21] | Evaluating scientific suitability of protocols as a prerequisite for Harm-Benefit Analysis (HBA). | Protocol details regarding model selection, experimental design, and intended inference space. | Sequential evaluation: 1. Construct Validity → 2. Internal Validity → 3. External Validity. | QUALITATIVE: A legitimacy decision (Scientifically suitable vs. unsuitable) prior to ethical review. |
| **2022 - Decision-Theoretic Framework (D-TF)**[22] | Quantifying the impact of predictive validity on R&D productivity; estimating the financial value of decision tools and validity-related information. | Quantitative Parameters: Predictive validity (correlation coefficient between tool score and clinical utility), Throughput (number of candidates), Selection thresholds, Base rate of clinical utility (rarity of good candidates), Costs and Profits. | Mathematical Modelling/Simulation: Treating R&D as a search process to calculate how changes in parameters (e.g., shifting validity from 0.5 to 0.6) affect the probability of success and economic return. | QUANTITATIVE: Positive Predictive Value (PPV); Net Present Value (NPV) of lifecycle cashflows per candidate. |
| **2022 - NAMs Scientific Confidence Framework (NAMs-SCF)**[23] | Establishing scientific confidence in New Approach Methodologies (NAMs) for regulatory use. | NAM data regarding protocol, human biology connection, technical reliability, and data integrity. | Evaluation against 5 essential elements: Fitness for Purpose, Human Biological Relevance, Technical Characterisation, Data Integrity, Independent Review. | QUALITATIVE: A determination of scientific confidence and "fitness for purpose" for regulatory application. |
| **2023 - Probability of Pharmacological Success (PoPS)**[24] | Informing "commit to clinical" decisions by integrating pharmacology and safety data into a single metric. | Exposure-response models; translational data (e.g., allometric scaling); quantified uncertainties in success criteria (intervention needs). | Simulation of virtual patient populations/trials using translational models and Monte Carlo simulations; results are checked against defined success criteria. | QUANTITATIVE: A single probability percentage (e.g., probability that ≥90% of patients achieve adequate pharmacology within safety limits). |
| **2024 - Preclinical Assessment for Translation to Humans (PATH)** [25] | Justifying the ethical and scientific rationale for launching early-phase trials | Mechanistic decomposition of the program into 9 steps; collect evidence (animal, in vitro, human) relevant to each link | Map studies to each step; rate confidence (High/Med/Low) based on effect, precision, and bias; explicitly score translational steps (model representativeness of target) | SEMI-QUANTITATIVE: Annotated PATH diagram; color-coded links Green/Yellow/Red; identifies “critical breaches”. |
| **2025 - Replacement Checklist**[26] | Confirming compliance with 3Rs (replacement, reduction and refinement). Ethical approval (Animal Welfare and Ethical Review Body, AWERB). | Research question; defined information sources (databases, experts, AI tools such as BimmoH) | Answer 6 non-prescriptive prompts; document why alternatives were rejected and why animal use is necessary | QUALITATIVE: Completed checklist providing an auditable record of Replacement due diligence |

# Supplementary References:

1. Tricco AC, Lillie E, Zarin W, O’Brien KK, Colquhoun H, Levac D, et al. PRISMA Extension for Scoping Reviews (PRISMA-ScR): Checklist and Explanation. Ann Intern Med. 2018;169:467–73. https://doi.org/10.7326/M18-0850

2. Cole V, Boutet M. ResearchRabbit (product review). J Can Health Libr Assoc J Assoc Bibl Santé Can. 2023;44:43–7. https://doi.org/10.29173/jchla29699

3. Zerhouni EA, Alving B. Clinical and Translational Science Awards: a framework for a national research agenda. Transl Res. 2006;148:4–5. https://doi.org/10.1016/j.lab.2006.05.001

4. Goodsaid FM, Frueh FW, Mattes W. The Predictive Safety Testing Consortium: A synthesis of the goals, challenges and accomplishments of the Critical Path. Drug Discov Today Technol. 2007;4:47–50. https://doi.org/10.1016/j.ddtec.2007.10.010

5. Lee D, Altar T. The Biomarkers Consortium: A public-private partnership of the Foundation for NIH. J Clin Oncol. 2007;25:14142–14142. https://doi.org/10.1200/jco.2007.25.18_suppl.14142

6. Hackam DG, Redelmeier DA. Translation of Research Evidence From Animals to Humans. JAMA. 2006;296:1727. https://doi.org/10.1001/jama.296.14.1731

7. Ouzzani M, Hammady H, Fedorowicz Z, Elmagarmid A. Rayyan—a web and mobile app for systematic reviews. Syst Rev. 2016;5:210. https://doi.org/10.1186/s13643-016-0384-4

8. Valentin J-P, Bialecki R, Ewart L, Hammond T, Leishmann D, Lindgren S, et al. A framework to assess the translation of safety pharmacology data to humans. J Pharmacol Toxicol Methods. 2009;60:152–8. https://doi.org/10.1016/j.vascn.2009.05.011

9. Wehling M. Assessing the translatability of drug projects: what needs to be scored to predict success? Nat Rev Drug Discov. 2009;8:541–6. https://doi.org/10.1038/nrd2898

10. Wendler A, Wehling M. Translatability score revisited: differentiation for distinct disease areas. J Transl Med. 2017;15:226. https://doi.org/10.1186/s12967-017-1329-y

11. Hooijmans CR, Rovers MM, de Vries RBM, Leenaars M, Ritskes-Hoitinga M, Langendam MW. SYRCLE’s risk of bias tool for animal studies. BMC Med Res Methodol. 2014;14:43. https://doi.org/10.1186/1471-2288-14-43

12. Smith MM, Clarke EC, Little CB. Considerations for the design and execution of protocols for animal research and treatment to improve reproducibility and standardization: “DEPART well-prepared and ARRIVE safely.” Osteoarthritis Cartilage. 2017;25:354–63. https://doi.org/10.1016/j.joca.2016.10.016

13. Hooijmans CR, De Vries RBM, Ritskes-Hoitinga M, Rovers MM, Leeflang MM, IntHout J, et al. Facilitating healthcare decisions by assessing the certainty in the evidence from preclinical animal studies. Minnerup J, editor. PLOS ONE. 2018;13:e0187271. https://doi.org/10.1371/journal.pone.0187271

14. Smith AJ, Clutton RE, Lilley E, Hansen KEA, Brattelid T. PREPARE: guidelines for planning animal research and testing. Lab Anim. 2018;52:135–41. https://doi.org/10.1177/0023677217724823

15. S. Ferreira G, Veening-Griffioen DH, Boon WPC, Moors EHM, Gispen-de Wied CC, Schellekens H, et al. A standardised framework to identify optimal animal models for efficacy assessment in drug development. Macleod MR, editor. PLOS ONE. 2019;14:e0218014. https://doi.org/10.1371/journal.pone.0218014

16. Storey J, Gobbetti T, Olzinski A, Berridge BR. A Structured Approach to Optimizing Animal Model Selection for Human Translation: The Animal Model Quality Assessment. ILAR J. 2021;62:66–76. https://doi.org/10.1093/ilar/ilac004

17. Gurusamy KS, Moher D, Loizidou M, Ahmed I, Avey MT, Barron CC, et al. Clinical relevance assessment of animal preclinical research (RAA) tool: development and explanation. PeerJ. 2021;9:e10673. https://doi.org/10.7717/peerj.10673

18. Emmerich CH, Gamboa LM, Hofmann MC, Bonin-Andresen M, Arbach O, Schendel P, et al. Improving target assessment in biomedical research: the GOT-IT recommendations. Nat Rev Drug Discov. Nature Publishing Group UK London; 2021;20:64–81.

19. Ochoa D, Hercules A, Carmona M, Suveges D, Gonzalez-Uriarte A, Malangone C, et al. Open Targets Platform: supporting systematic drug-target identification and prioritisation. Nucleic Acids Res. 2021;49:D1302–10. https://doi.org/10.1093/nar/gkaa1027

20. Bespalov A, Bernard R, Gilis A, Gerlach B, Guillen J, Castagne V, et al. Introduction to the EQIPD quality system. Elife. eLife Sciences Publications, Ltd; 2021;10:e63294.

21. Eggel M, Würbel H. Internal consistency and compatibility of the 3Rs and 3Vs principles for project evaluation of animal research. Lab Anim. 2021;55:233–43. https://doi.org/10.1177/0023677220968583

22. Scannell JW, Bosley J, Hickman JA, Dawson GR, Truebel H, Ferreira GS, et al. Predictive validity in drug discovery: what it is, why it matters and how to improve it. Nat Rev Drug Discov. 2022;21:915–31. https://doi.org/10.1038/s41573-022-00552-x

23. Anna J. van der Zalm, Anna J. van der Zalm, João Barroso, José G. Barroso, Patience Browne, Patience Browne, et al. A framework for establishing scientific confidence in new approach methodologies. Arch Toxicol. 2022; https://doi.org/10.1007/s00204-022-03365-4

24. Chen C, Zhou X, Lavezzi SM, Arshad U, Sharma R. Concept and application of the probability of pharmacological success (PoPS) as a decision tool in drug development: a position paper. J Transl Med. 2023;21:17. https://doi.org/10.1186/s12967-022-03849-y

25. Kimmelman J, Bodilly Kane P, Bicer S, Carlisle BG. Preclinical assessment for translation to humans: The PATH approach for assessing supporting evidence for early-phase trials and innovative care. Med N Y N. 2024;5:1227–36. https://doi.org/10.1016/j.medj.2024.07.014

26. Dukes JP, Beale A, Camp C. Reviewing Current Guidance for the ‘R’ of Replacement and Rethinking it with the ‘Replacement Checklist.’ Altern Lab Anim. 2025;53:72–83. https://doi.org/10.1177/02611929251319265
